# A Jumble of Hospital Horrors
*St. Bernard's: The Romance of a Medical Student. By Æsculapius Scalpel. Swan Sonnenschein and Co.


**Published:** 1887-12-24

**Authors:** 


### Turning over New Leaves.

### A JUMBLE OF HOSPITAL HORRORS.*

This is a book which, as the author is evidently in earnest, ought not to have been published anonymously. It possesses no artistic merit, it is replete with evidences of questionable taste, it is a jumble of horrors, but it claims notice as a vigorous, if unscrupulous, attack upon institutions which most members of the community have been taught to regard with pride and confidence. The manlier and more satisfactory course for a reformer so ardent would have been to lead his attack in the open, rather than to adopt the tactics of the dynamiter and the assassin. If the hospitals are the horrible torture-houses he depicts, by all means let the sun shine upon their dark and cruel deeds ; but we confess to a suspicion of the man with a lantern who moves about masked, and while affecting to cast light upon the scene, is careful to keep his own features in shadow.

That there is much which is capable of improvement, alike in the administration of hospitals and in the social amenities of their inner life, we are quite free to admit; but the man who knows most of their defects generally has a sufficient acquaintance with their merits and a sufficient sense of their indispensability to induce him to temper criticism with discretion. If we are to draw any conclusion at all from the teaching of "St. Bernard's," it is that the very atmosphere of our hospitals is leprous, and their whole environment revolting. In them, neither decency nor humanity finds a place. Everybody, from the hall porter and the casualty nurse to the senior surgeons and physicians, practises systematic deception and cruelty ; patients are betrayed, hacked, and murdered for the benefit—aye, for the amusement—of the students ; and if a sufferer should chance to get well, he may thank his stars he was not an interesting "subject," or his carcase would as certainly have been thrown to the "schools," as Reynard's is to the hungry pack which has hounded him down.

In dealing with the scenes he describes, the author makes the most of those details of horror which of necessity abound. We are spared nothing : he revels in the loathsome ; he garnishes it with his luxuriant fancy, and when plain truth appears ineffective he seasons it with fable. Witness the childish episode of the night spent in the dissecting-room—worthy of a schoolboy's ghost story—and the physician who is made to do away with his wife by slow poison ! We do not, of course, say it is impossible for a physician to be a barbarian, but in a book not avowedly fictitious—on the contrary, ostentatiously claiming to depict truth—it is neither honest nor sensible to hold up an odious murderer as a type of the hospital physician.

Yet while our author, who affects to be, and probably is, a medical man, publicly vivisects without compunction the body of his Alma Mater in this fashion, he boasts so little sense of humour that he proceeds with a tirade against vivisection generally, and does not hesitate to introduce into his pages the names of living men whose labours have conferred undying benefit upon mankind, in order that he may cover them with reproach and obloquy. Had he confined himself to denouncing the repetition of experiments the results of which have been already placed beyond question, or to inveighing against the mere torturing of living animals by unauthorised inexperts, his strictures would have been as praiseworthy as commonplace, for who does not heartily agree? They could hardly have been too severe. Vivisection will never be more than tolerated by the public at large—it will never appeal to general sympathy, and we can not only forgive, but commend the impulse which condemns it. Yet we look for somewhat keener perception, if not for greater generosity of judgment in one who has received a medical training ; and whether he allows the investigator the ordinary sensibilities of humanity, or pictures him as possessed of a devil of cruelty, our author is scarcely abreast of the times if he fails to perceive, and to estimate at something of their real value, the splendid achievements of contemporary science.

The story, such as it is, makes us acquainted with a "hero," a medical student, who, after starting with noble aspirations, betrays his better nature, sinks to the level of the rowdy insensate company he consorts with, suddenly repents and then, oddly enough from a literary point of view, disappears entirely, only to be found again in the last chapters of the book, by which time he is purged of his sins, and has recovered the pristine nobility of his character. He is unearthed by—and ultimately marries—a rich heiress who has been in love with him throughout, and who, at his instigation, and that of her own enthusiasm, devotes her wealth to the scattering of small hospitals of fifty beds each about the metropolis, where we are led to believe that an infusion of lady doctors and the banishment of students work miracles in behalf of the sick, who are happy ever after. How the lady doctors are to be trained when the hospitals have ceased to be utilized for teaching we are not told, but perhaps the author believes that the feminine faculty of intuition will render training unnecessary.

We are inclined to describe this book as a libel, but a libel neither subtle nor clever enough to be very dangerous. Were it possible for the book to do harm, it is the poor who would be the chief sufferers, and they, we are assured, are the author's chief concern. Once instil into the minds of those who support hospitals, and of those who use them, the belief that these institutions are administered without regard to the feelings and well-being of the patients, and we are confronted with grave social problems which the author of "St. Bernard's" could lend us no help to solve. It is the more to be regretted that he writes in so reckless a fashion, because there are possibilities of occasional agreement with what he says, had he said it temperately and with discrimination. We may remark of this book that its publication is not only a crime, it is a blunder, because it is calculated to arrest reform. Instead of arousing managers to a sense of shortcoming, these sweeping diatribes—these assertions of wholesale mutilation and murder—this inability to see any good because there is some imperfection, which would be calculated to raise a smile were the subject less lugubrious, can now only arouse resentment. The work of our hospitals is not to be denounced because here and there its perfection is marred by the errors and heartlessness of individual workers, any more than criticism is to be deprecated because of the eccentricities of the author of "St. Bernard's."

No doubt there is great room for improvement in the system which places very young, and, except in regard to the text-books of their profession, very uninformed, men in the powerful positions of house physicians and house surgeons, and insists upon their retirement after a few months of office, in order to give place to their juniors. These offices might be held advantageously by maturer men, and the holders need to be brought into closer alliance with the institution itself, and as a consequence into somewhat more complete subjection. It is regrettable to think that too often the only qualification needed for posts so responsible is merit in the schools, that personal considerations may be lost sight of, and that there must be in all but exceptional cases an absence of the tact in dealing with other people, and the consideration for their feelings, which only experience and knowledge of the world can bring. At the same time, when we take into account the shortcomings of the system, we can only marvel

* St. Bernard's: The Romance of a Medical Student. By Æsculapius Scalpel. Swan Sonnenschein and Co.

there is so much to commend in its exponents. Regarded from a patient's point of view, their very errors "lean to virtue's side." Witness the extravagant diets, the ever-increasing "extras," the prevalent belief among young medical men that nothing is to be stinted—that no plea of economy is to avail. Is it not often said, and said reproachfully, by visitors and subscribers, that the poor get for nothing in our hospitals such costly comforts and unremitting attentions as the middle classes look for vainly in the hour of their own sickness? It is simple nonsense for anyone—particularly for a medical man—to object that the patient is asked to afford, in exchange for all these advantages, the only return in his power—the opportunity for clinical teaching.

Undoubtedly, an ideal hospital is an organisation religious and philanthropic, and to those who seek to trace the golden threads of love and pity running bright and unbroken through the whole fabric, there must be occasional disappointment; but we know of no hospital which is even distantly related to our author's imaginary institution, where all the authorities are corrupt, and boast not a rag of piety between them. When the physicians and surgeons of hospitals do really take to murdering their wives, and become as brutal generally as the staff of St. Bernard's is represented to be, then indeed the case will be hopeless; but so long as the institutions command the services of the very cream of the profession, and so long as the lay and religious elements in their management are maintained unimpaired, the public may rest assured that the history of St. Bernard's will not cease to be apocryphal. Nor need we blush over our own simplicity if we continue to believe that Crabbe's well-known description may be applied to those noble institutions which an eminent canon of the present day has said are "among the fairest flowers in the garland of our humanity":

"Fevers and chronic ills, corroding pains,
Each accidental mischief man sustains;
Fractures and wounds, and withered limbs and lame,
With all that, slow or sudden, vex our frame,
Have here attendance—here the sufferers lie,
Where love and science every aid apply,
And healed, with rapture live, or soothed by comfort, die."

